# Global burden and trends of *Klebsiella pneumoniae* infection, 1990–2021: insights from the global burden of disease study

**DOI:** 10.3389/fpubh.2025.1630262

**Published:** 2025-10-23

**Authors:** Jiangang Ju, Xiaona Liu, Qingqing Chen, Jian Wang, Linfeng Shen

**Affiliations:** ^1^Department of Respiratory and Critical Care Medicine, Zhejiang University School of Medicine Second Affiliated Hospital Linping Campus, Hangzhou, Zhejiang, China; ^2^Department of General Practice, Yunhe Street Community Health Service Center, Hangzhou, Zhejiang, China

**Keywords:** *Klebsiella pneumoniae*, global burden of disease, epidemiology, trend, average annual percentage changes

## Abstract

**Background:**

*Klebsiella pneumoniae (KP),* a prominent member of the Enterobacteriaceae family, is recognized as an opportunistic pathogen responsible for a variety of diseases. Despite its significant threat to public health, there is a lack of epidemiological information concerning the burden of *KP* infection in the lower respiratory tract.

**Methods:**

Age-standardized rates (ASR) of disability-adjusted life-years (DALYs) and deaths rates (ASDRs) attributed to *KP* infection were obtained from Global Burden of Disease (GBD) 2021, stratified by sex, age, socio-demographic Index (SDI) quintiles and seven super regions. We also calculated the average annual percentage changes (AAPCs) of ASR-DALYs and ASDRs for *KP* infection using the Joinpoint regression analysis to evaluate the trend of disease burden.

**Results:**

In 2021, the global ASR-DALYs and ASDRs attributable to *KP* infection were 124.4 and 2.68 per 100,000, with AAPCs of −3.23% and −2.42%, respectively. The highest burden of ASR-DALYs was observed in children under 5 years of age, with a rate of 775.75 per 100,000 (95% uncertainty interval [UI]: 601.07 to 973.76), while the highest ASDRs were found in individuals over 70 years of age, with a rate of 18.05 per 100,000 (95% UI: 15.84–19.70). Notably, there were significant increasing trends in DALYs and death rates due to *KP* infection in Central Europe, Eastern Europe, and Central Asia across all age groups above 15 years, with the most pronounced increase observed in individuals over 70 years of age, characterized by AAPCs of 0.85% (95% confidence interval [CI]: 0.64 to 1.05) and 1.00% (95% CI: 0.85–1.17), respectively.

**Conclusion:**

Over the past 32 years, the global burden of *KP* infection in the lower respiratory tract has generally declined, but it has increased among the older population in Central/Eastern Europe and Central Asia. This rise is likely due to inappropriate antibiotic use, widespread antimicrobial resistance, emerging virulent and multidrug-resistant strains, and an aging population, highlighting the need for vigilant monitoring and intervention measures.

## Introduction

*Klebsiella pneumoniae (KP),* a Gram-negative rod-shaped member of the *Enterobacteriaceae* family, colonizes the human gut and oropharynx asymptomatically but transforms into a formidable pathogen under immunosuppressed conditions (1, 2). *KP* infection in the lower respiratory tract was initially associated with community-acquired pneumonia (CAP) in populations of diabetics and alcoholics. However, since the advent of the antibiotic era, it has evolved into a major healthcare-associated pathogen (HAP). Currently, *KP* is the causative agent of severe nosocomial infections, including pneumonia, urinary tract infections (UTIs), cystitis, surgical wound infections and life-threatening infections like endocarditis and septicemia. These infections predominantly affect inpatients and immunocompromised individuals, especially those who have been using antibiotics for an extended period or are undergoing invasive medical procedures (3).

*KP* infection presents a significant public health threat due to its strong pathogenic potential and close association with multidrug resistance. The bacterium rapidly disseminates in healthcare settings through the production of antibiotic resistance genes, particularly carbapenemases (e.g., KPC, NDM, VIM, OXA-48-like), leading to difficult-to-treat infections (3, 4). In China, *KP* infection accounts for 11.9% of ventilator-associated and ICU-acquired pneumonia cases, with severe infection rates in neonatal units ranging from 18% to 68% (5, 6). The escalating threat posed by *KP* infection is further intensified by the global rise in extended-spectrum *β*-lactamase (*ESBL*)-producing and carbapenem-resistant (CR) strains (7–9). *ESBL*-producing *Klebsiella pneumoniae* (*ESBL-KP*) are relatively prevalent worldwide, with an average prevalence rate in humans of 32.7% (10). Furthermore, carbapenem-resistant *Klebsiella pneumoniae* (CRKP) also has been increasingly reported in both healthcare associated infection and environment during recent years worldwide (11, 12). In GBD 2019 Antimicrobial Resistance study, *KP* was associated with a greater number of deaths (1,105,000) and a burden of years of life losts (YLLs) of 31.4 million (13). The burden of *KP* infection is associated with more deaths and YLLs burden than *Streptococcus Pneumoniae* or tuberculosis (14).

However, despite the significant clinical impact of *KP* infection, comprehensive global data on the burden and trends of *KP* infection in the lower respiratory tract remain fragmented (15). Using the GBD 2021 database, we examined the DALYs and death burdens associated with *KP* infection in the lower respiratory tract. This study provides a comprehensive analysis of *KP* infection trends and variations in both temporal and spatial dimensions. Specifically, our study has the following objectives: (1) a descriptive and trend analysis of *KP* infection burden at global and regional levels, and (2) an investigation of spatial and temporal variations in *KP* infection patterns. This approach offers a more complete understanding of the global burden of *KP* infection and their trends over the last three decades.

## Materials and methods

### Data sources and disease definition

This research is a retrospective observational study that analyzes the disease burden using secondary data from the GBD 2021 database, a comprehensive collaborative initiative led by the Institute for Health Metrics and Evaluation (IHME) at the University of Washington (16, 17). As an open-access health data repository, the GBD database systematically quantifies the health loss attributed to 371 diseases and injuries and 88 risk factors across 204 countries and territories from 1990 to 2021. It serves as a critical resource for global health research and evidence-based policymaking, with continuous updates since its inception in 1990. Our analysis specifically utilized *KP* infection in the lower respiratory tract epidemiology metrics from this global dataset, accessible via the IHME official portal.^1^ Estimates of these metrics were calculated using the Bayesian hierarchical meta-regression tools including the Cause of Death Ensemble model (CODEm) for estimating fatal outcomes and YLLs, and DisMod-MR 2.1, a Bayesian meta-regression tool for evaluating nonfatal health loss (16). Detailed data sources and model methods were reported in GBD 2021 (16). In addition, GBD study employs a multi-tiered geographic classification system comprising seven super-regions: (1) Sub-Saharan Africa; (2) North Africa and Middle East; (3) South Asia; (4) Southeast Asia, East Asia and Oceania; (5) Latin America and Caribbean; (6) Central Europe, Eastern Europe and Central Asia; and (7) High-income regions. The SDI serves as a composite index of development status, showing a strong correlation with health outcomes. The 204 countries and territories were grouped into five SDI quintiles: low-SDI, low-middle-SDI, middle-SDI, high-middle-SDI, and high SDI regions (18). The age groups were divided into five categories: under 5, 5–14, 15–49, 50–69, and over 70 years.

The *KP* infection in the lower respiratory tract is identified by the International Classification of Diseases, Ninth Revision (ICD-9) codes (482.0) and ICD-10 codes (J15.0) for diagnosis (13).

### Statistical analysis

The data used Age-standardized rates of disability-adjusted life-years (ASR-DALYs, per 100,000 population) and age-standardized death rates (ASDRs, per 100,000 population) as main indicators to measure the disease burden. The metric of disability-adjusted life years (DALYs) is calculated through dual components: years of life lost (YLLs) derived from premature mortality (calculated as deaths multiplied by standard life expectancy at death age) and years lived with disability (YLDs) determined by multiplying case numbers with condition-specific disability weights and duration of impairment (16). Death rates quantify the number of deaths in a population over a designated time or area, illustrating the ratio of deaths to the total population. The GBD is processed using standardized algorithms (e.g., CODEm, ST-GPR, and DisMod-MR) to address issues such as incompleteness, misclassification, and stochastic variability. To further enhance data reliability, the framework not only generates point estimates but also calculates the 95% uncertainty interval. The 95% UI was calculated from 1,000 simulated samples, using the 2.5th and 97.5th percentiles to determine its bounds. Using the World Health Organization World Standard Population Distribution, age-standardized rates (ASR) were produced to allow for comparisons across groups with diverse age demographic compositions, which were calculated according to the following formula: $\mathrm{ASR}=\frac{\sum_{i=1}^{n}s_{i}R_{i}}{\sum_{i=1}^{n}R_{i}}\times 100,000.$

(*n* represents the number of age groups; *S_i_*: represents the standard population size of the *i*-th age group; *R_i_*: represents the actual age-specific rate of the *i*-th age group). To estimate ASR trends over time, we used Joinpoint regression software (version 4.8.0.1, National Cancer Institute) to describe the change trends of *KP* infection from 1990 to 2021. Since data are time series and may exhibit autocorrelation, we applied an autocorrelation correction in the Joinpoint regression analysis (19). The annual percentage change (APC) with its 95% confidence interval (CI) indicates each trend segment (20). Based on the weighted average of the segmented annual percentage change over a specified interval, the average annual percentage change (AAPC) with its 95% CI meaning annual was calculated to explore the average change rate of the ASR-DALYs and ASDRs of *KP* infection during 1990–2021. An upward trend is indicated when both the AAPC and the lower boundary of the 95% CI are positive, whereas a downward trend is suggested when both the AAPC and the upper boundary of the 95% CI are negative. All statistical analyses and data visualizations were conducted using R software (version 4.4.1). For the trend analysis, a *p*-value of less than 0.05 was considered statistically significant.

## Results

### Global burden trends

**Figure 1:**
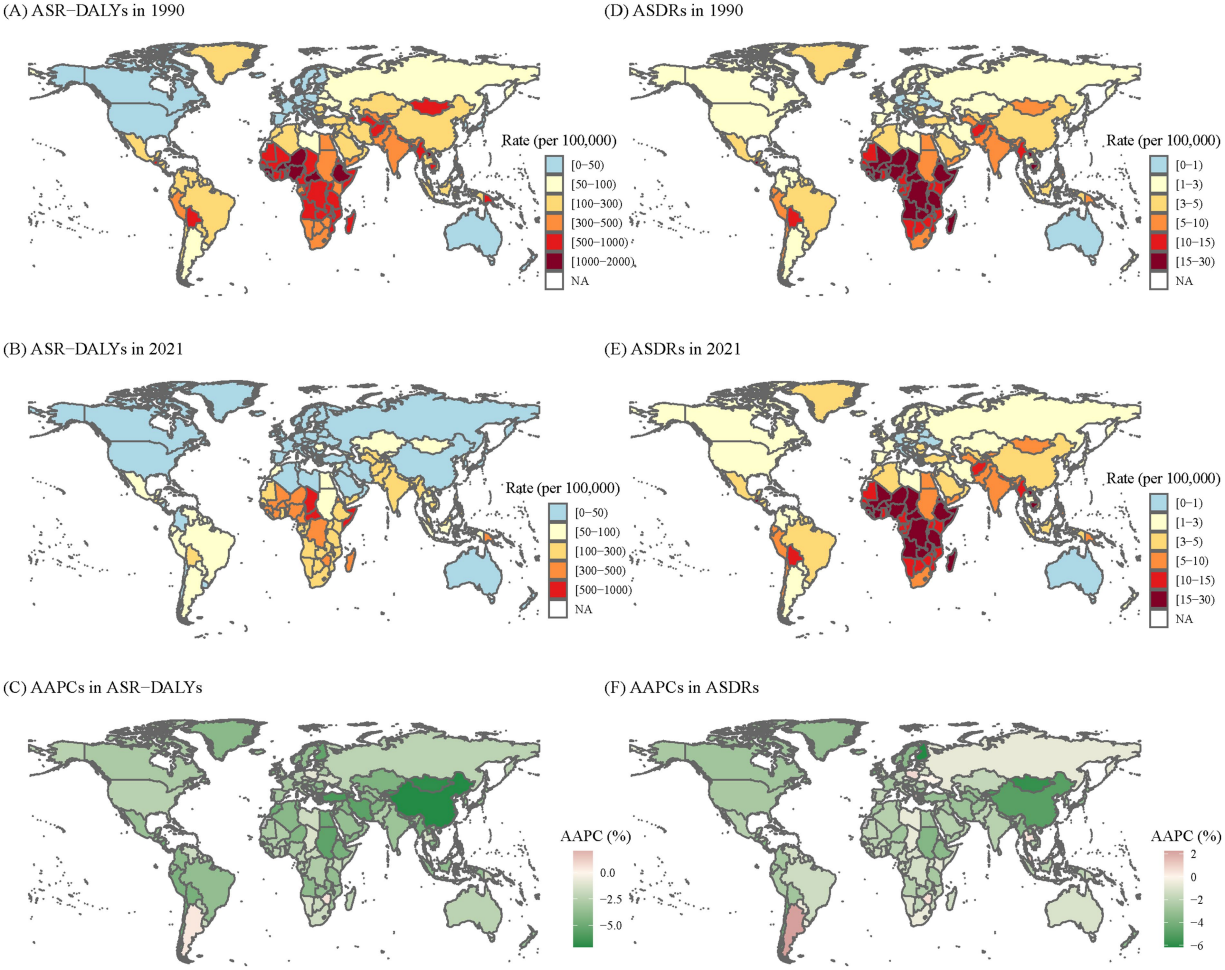
The global disease burden of KP infection in 204 countries and territories. **(A)** ASR-DALYs of *KP* infection in 1990. **(B)** ASR-DALYs of KP infection in 2021. **(C)** AAPCs of ASIR. **(D)** ASDRs of KP infection in 1990. **(E)** ASDRs of KP infection in 2021. **(F)** AAPCs of ASDRs. *KP*, *Klebsiella pneumoniae*; ASR-DALYs, age-standardized disability-adjusted life years; ASDR, age-standardized death rate; AAPCs, average annual percentage changes.

As shown in Table 1 and Figure 1, from 1990 to 2021, the global ASR-DALYs per 100,000 of *KP* infection decreased from 339.03 (95%UI: 295.55–387.42) to 124.4 (95%UI:104.09–147.23), with the AAPCs of −3.19 (95% CI: −3.21 to −3.17). ASDRs per 100,000 population also exhibited a downward trend during this period, declining from a rate of *KP* infection-related deaths at a rate of approximately from 5.58 (95% UI: 5.06–6.18) in the year 1990 to around 2.68 (95% UI: 2.37–2.99) in 2021, with the AAPCs of −2.33 (95% CI: −2.36 to −2.29). In 1990 and 2021, men experienced marginally higher ASR-DALYs and ASDRs burdens compared to women. Despite a significant decrease of ASR-DALYs in the younger than 5-year age group (AAPC = −4.03, 95% CI: −4.07 to −4.00), the burden remained the highest in 2021 (775.75 per 100,000, 95% UI: 601.07–973.76). In addition, for those over 70, ASDRs were on the decline, yet they experienced the greatest burden in 2021 compared to other age groups (18.05 per 100,000, 95% UI: 15.84–19.7).

**Table 1:** The ASR-DALYs and ASDRs and their AAPCs of *KP* infection worldwide in 1990 and 2021.

Group	ASR-DALYs, per 100,00		AAPCs%, 95CI	ASDRs, per 100,000		AAPCs%, 95CI
	1990	2021		1990	2021	
Global	339.03 (295.55–387.42)	124.4 (104.09–147.23)	−3.19 (−3.21 to −3.17)^*^	5.58 (5.06–6.18)	2.68 (2.37–2.99)	−2.33 (−2.36 to −2.29)^*^
Gender						
Female	326.49 (282.81–376.59)	112.94 (94.24–133.34)	−3.38 (−3.42 to −3.35)*	5.13 (4.56–5.75)	2.33 (2.01–2.63)	−2.52 (−2.55 to −2.49)*
Male	353.65 (306.8–405.76)	136.7 (113.57–162.58)	−3.01 (−3.02 to −2.99)*	6.24(5.66–6.84)	3.14 (2.82–3.51)	−2.19 (−2.22 to −2.16)*
Age group						
<5 years	2768.15 (2373.56–3223.48)	775.75 (601.07–973.76)	−4.03 (−4.07 to −4)^*^	31.07(26.64–36.2)	8.7 (6.73–10.94)	−4.04 (−4.07 to −4)^*^
5–14 years	74.13 (60.65–89.23)	33.2 (27.54–40.28)	−2.55 (−2.61 to −2.5)^*^	0.9 (0.73–1.08)	0.4 (0.33–0.49)	−2.54 (−2.59 to −2.48)^*^
15–49 years	31.33 (27.44–35.51)	23.36 (20.23–26.55)	−0.96 (−1.01 to −0.9)^*^	0.52 (0.46–0.59)	0.41 (0.35–0.46)	−0.82 (−0.87 to −0.76)^*^
50–69 years	99.05 (90.09–109.36)	70.43 (63.95–76.67)	−1.05 (−1.09 to −1.02)^*^	3.27 (2.98–3.61)	2.35 (2.13–2.57)	−1.04 (−1.07 to −1)^*^
70+ years	311.58 (280.7–339.78)	238.43 (211.95–259.8)	−0.83 (−0.88 to −0.79)*	22.23 (19.72–24.26)	18.05 (15.84–19.7)	−0.64 (−0.69 to −0.58)^*^
SDI ranks						
High SDI	38.52 (36.28–40.64)	15.67 (14.38–16.5)	−2.83 (−2.9 to −2.74)^*^	1.68 (1.52–1.77)	0.82 (0.71–0.89)	−2.25 (−2.33 to −2.15)^*^
High-middle SDI	128.68 (115.19–145.87)	27.88 (25.87–29.99)	−4.83 (−4.91 to −4.76)^*^	2.38 (2.19–2.61)	1.07 (0.96–1.17)	−2.53 (−2.61 to −2.43)*
Middle SDI	265.1 (236.73–293.62)	71.01 (63.51–80.2)	−4.2 (−4.24 to −4.15)^*^	5 (4.55–5.43)	2.19 (1.98–2 0.4)	−2.66 (−2.69 to −2.62)^*^
Low-middle SDI	460.79 (401.72–526.66)	161.77 (138.32–186.96)	−3.34 (−3.37 to −3.3)^*^	7.9 (7.03–8.79)	4.12 (3.63–4.6)	−2.08 (−2.12 to −2.05)^*^
Low SDI	812.24 (684.71–964)	299.64 (246.27–366.85)	−3.17 (−3.2 to −3.14)^*^	14.75 (12.94–16.81)	7.48 (6.47–8.56)	−2.16 (−2.19 to −2.14)^*^
GDB super regions						
North Africa and Middle East	267.88 (233.16–322.81)	65.53 (56.4–75.17)	−4.44 (−4.48 to −4.39)^*^	4.57 (4.07–5.24)	1.92 (1.68–2.14)	−2.73 (−2.77 to −2.67)^*^
Sub-Saharan Africa	827.55 (691.41–994.32)	338.87 (272.96–413.14)	−2.84 (−2.86 to −2.81)^*^	15.95 (13.99–18.17)	9.09 (7.78–10.36)	−1.79 (−1.81 to −1.78)^*^
Central Europe, Eastern Europe, and Central Asia	162.69 (151.36–175.8)	65.33 (57.86–73.48)	−2.86 (−2.93 to −2.78)^*^	2.33 (2.19–2.49)	1.35 (1.24–1.45)	−1.64 (−1.73 to −1.54)^*^
Latin America and Caribbean	212.64 (196.37–230.53)	71.38 (63.01–80.98)	−3.47 (−3.56 to −3.4)^*^	4.78 (4.48–5.12)	2.56 (2.27–2.81)	−1.95 (−2.05 to −1.86)*
Southeast Asia, East Asia, and Oceania	278.45 (246.79–314.29)	60.5 (52.94–68.75)	−4.83 (−4.86 to −4.79)^*^	5.05 (4.54–5.55)	1.78 (1.58–1.99)	−3.34 (−3.41 to −3.29)^*^
South Asia	435.16 (369.91–498.04)	148.18 (127.17–169.84)	−3.45 (−3.54 to −3.37)^*^	7.33 (6.44–8.22)	3.65 (3.22–4.1)	−2.19 (−2.25 to −2.12)*
High-income	34.6 (32.6–36.11)	15.36 (13.94–16.21)	−2.53 (−2.64 to −2.44)^*^	1.58 (1.43–1.67)	0.82 (0.71–0.89)	−2.03 (−2.13 to −1.94)^*^

^*^Asterisk indicate *p* < 0.05.

ASR-DALYs, age-standardized disability-adjusted life years; ASDR, age-standardized death rate; AAPCs, average annual percentage change; 95% CI, 95% confidence interval; SDI, sociodemographic index.

### Trends of burdens among SDI quintiles

As shown in Table 1, according to SDI quintiles, the ASR-DALYs and ASDRs of *KP* infection exhibited a downward trend during the study period, but the burden varied considerably among different SDI regions. In 2021, the highest burden of ASR-DALYs (299.64 per 100,000, 95%UI: 246.27–366.85) and ASDRs (7.48 per 100,000, 95%UI: 6.47–8.56) both in low SDI regions; the lowest burden of ASR-DALYs (15.67 per 100,000, 95%UI: 14.38–16.5) and ASDRs (0.82 per 100,000, 95%UI: 0.71–0.89) attributable to *KP* infection both in high SDI regions. Among five SDI regions, the region where the ASR-DALYs decreased most significantly were in the high-middle SDI regions, with AAPCs of −4.83% (95% CI: −4.91 to −4.76).

As shown in Supplementary Table 1 and Supplementary Figure 1, the DALYs and death rates of *KP* infection in all age groups among SDI quintiles experienced a notable decline, with a more pronounced decline in younger than 5-year age group (AAPCs of DALYs among SDI quintiles were −5.72, −7.93%, −5.91, −4.49%, and −4.19%, respectively; AAPCs of death rates among SDI quintiles were −5.72, −7.94%, −5.91, −4.49%, and −4.20%, respectively).

### Trends of burdens among super regions

As shown in Table 1 and Supplementary Figure 2, among the seven super regions defined by GBD, in 2021, the regions with the highest ASR-DALYs and ASDRs of *KP* infection were both in sub-Saharan Africa (338.87 per 100,000, 95% UI: 272.96–413.14; 9.09 per 100,000, 95%UI: 7.78–10.36, respectively); and the regions with the lowest ASR-DALYs and ASDRs were both in high-income regions (15.36 per 100,000, 95% UI: 13.94–16.21; 0.82 per 100,000, 95% UI: 0.71–0.89). Furthermore, the most decrease for ASR-DALYs and ASDRs of *KP* infection during the study period had been among the Southeast Asia, East Asia, and Oceania, with AAPCs of −4.83% (95% CI: −4.86 to −4.79) and −3.34% (95% CI: −3.41 to −3.29).

**Figure 2:**
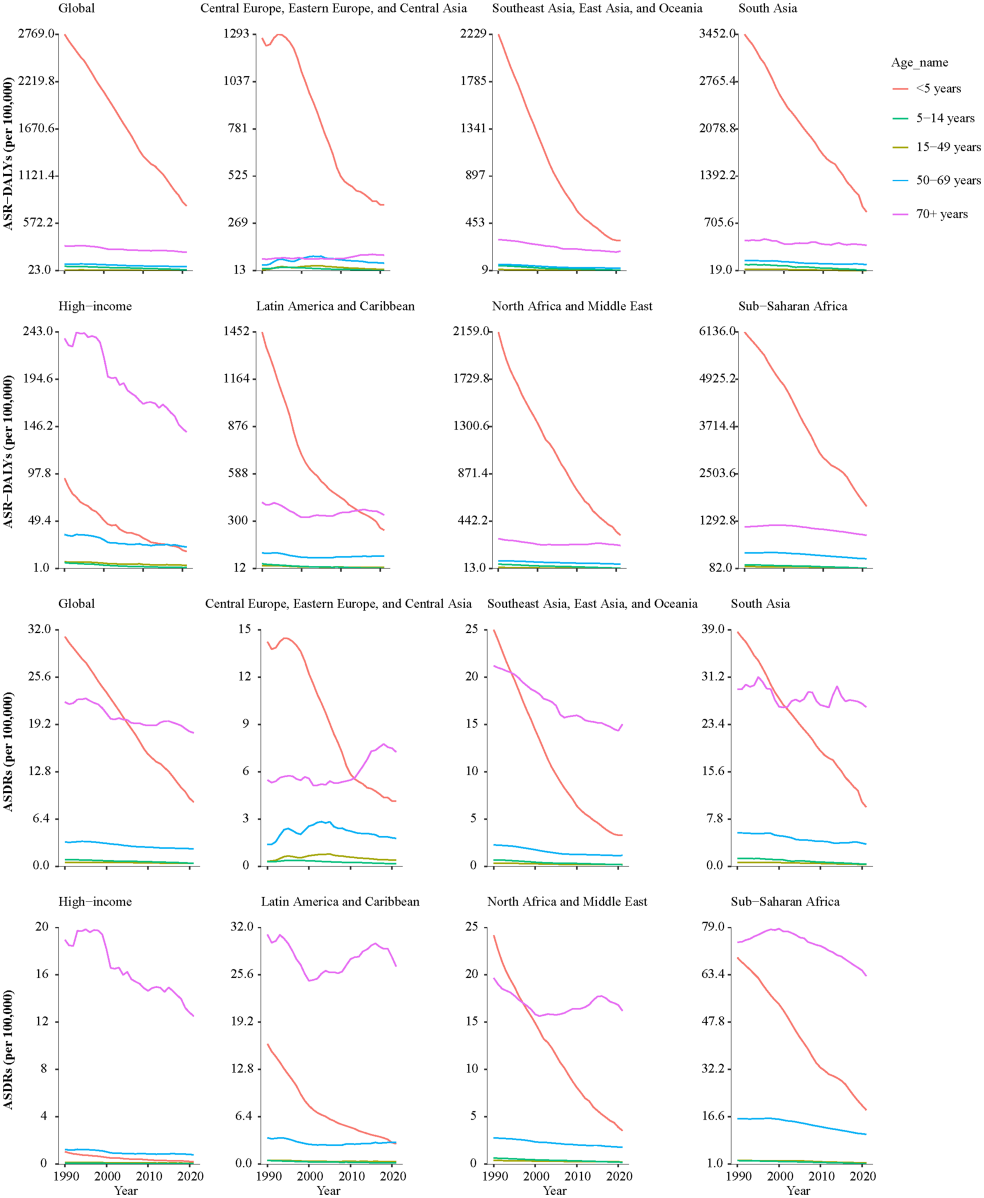
The AAPCs of ASR-DALYs and ASDRs of *KP* infection across all super regions in different age groups from 1990 to 2021. *KP*, *Klebsiella pneumoniae*; ASR-DALYs, age-standardized disability-adjusted life years; ASDR, age-standardized death rate; AAPCs, average annual percentage changes.

As shown in Figure 2 and Supplementary Table 2, from 1990 to 2021, the DALYs and death rates of *KP* infection exhibited declining trends across all super regions, with the exception of Central Europe, Eastern Europe, and Central Asia. In Central Europe, Eastern Europe, and Central Asia, the DALYs and death rates in all age groups older than 15 years had a significant increase, with AAPCs of DALYs were 0.35% (95% CI: 0.15–0.55), 0.82% (95% CI: 0.62–1.04) and 0.85% (95% CI: 0.64–1.05), while AAPCs of death rates were 0.63% (95% CI: 0.41–0.87), 0.92% (95% CI: 0.7–1.18) and 1.00% (95% CI: 0.85–1.17), respectively.

### Relationship between the AAPCs of bureden with SDI scores

**Figure 3:**
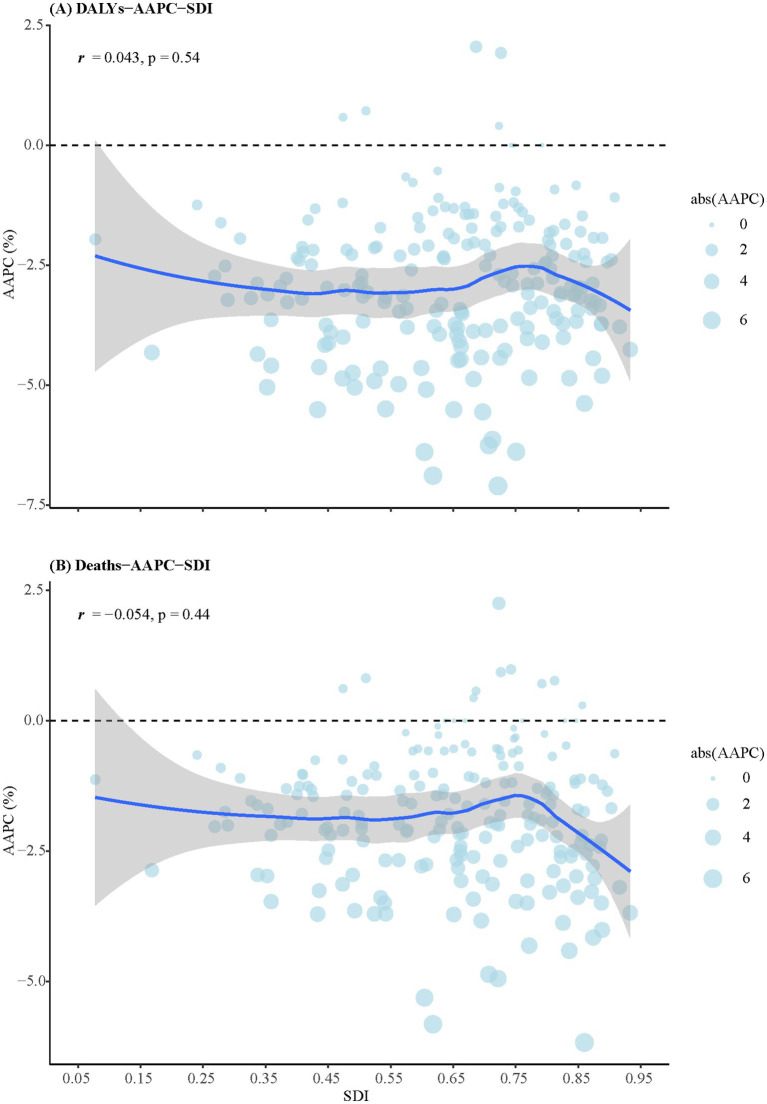
The relationships between AAPCs of DALYs and death rates with SDI scores. **(A)** DALYs. **(B)** Death rates. DALYs, disability-adjusted life years; SDI, sociodemographic index; AAPCs, average annual percentage changes.

Figure 3 shows the relationships between AAPC of DALYs (Figure 3A) and death rates (Figure 3B) with SDI scores. There was no significant correlation between the AAPC of DALYs and SDI scores (*r* = 0.043, *p* = 0.53), nor between AAPC of death rates and SDI scores (*r* = −0.054, *p* = 0.44).

## Discussion

In this study, we systematically delineated the comprehensive epidemiological patterns and temporal dynamics of *KP* infection burden in the lower respiratory tract—including ASR-DALYs and ASDRs—across sexes, age groups SDI categories and geographic regions. Our study suggested that the worldwide decrease in ASR-DALYs and ASDRs of *KP* infection from 1990 to 2021. The decline in the global disease burden of *KP* infection is associated with multiple factors, including significantly strengthened hospital infection control measures (21), optimized antimicrobial stewardship and resistance management (22, 23), advances in medical technology and diagnostic technology (24, 25), enhanced public health interventions (26), and the synergistic effects of regional differences and global prevention and control strategies (25). However, compared to GBD level 3 underlying causes of death, five leading pathogens-related deaths, with *KP* being among them, have been the second leading cause of death globally in 2019 (13), suggesting that prevention and control of the disease burden of *KP* infection will be necessary and significant.

The global burden of *KP* infection varied significantly across different SDI levels. Our findings showed that the burden of *KP* was lowest and declining in high SDI regions, similar to trends in other infectious diseases like tuberculosis and malaria. This can be attributed to several factors, including stringent antibiotic usage protocols (encompassing precise drug selection and the restriction of broad-spectrum antibiotics) (27–29), advanced healthcare systems, and robust infection control measures (such as hand hygiene, contact isolation, and environmental disinfection) (27). Additionally, effective surveillance mechanisms (including accurate source tracing, outbreak prediction, and elucidation of transmission dynamics) (30), and significant investment in research and resources (for instance, the development of novel therapeutics and diagnostic technologies) play a crucial role (25). Although the burden of *KP* infection was also showing a downward trend in low SDI regions, it was still significantly heavier, particularly in sub-Saharan Africa. The results were similar to those found in previous studies (13, 31). Previous researches have suggested that the disproportionately high infection rates in low- and middle-income countries can largely be attributed to limited access to effective antimicrobials, fragile health systems, and insufficient prevention programs (32, 33). Furthermore, the frequent wars and conflicts in recent years have caused catastrophic damage to the public health system, directly leading to a significant increase in the burden of *KP* infections (25). Therefore, the United Nations Secretary-General has identified healthcare facilities as a critical area requiring urgent attention to achieve the Sustainable Development Goals (SDGs) by 2030 (34). In addition, targeted interventions should be designed to strengthen antimicrobial stewardship, enhance healthcare infrastructure, and resistance gene prevalence characteristics in high-burden areas (e.g., sub-Saharan Africa). However, there was no notable correlation between AAPC of DALYs or death rates and SDI scores. Disparities may arise from regional differences in infection control, healthcare access, antimicrobial resistance (AMR) issues, local antibiotic practices, diagnostic capacity, and healthcare reporting, as well as regional risk factors like demographics, comorbidities, and environmental conditions, affecting detection and reporting of *KP* infections independently of SDI (35, 36).

Our study found the burden of ASDRs in the age group older than 70 remained higher. *KP* is known for its high virulence and antimicrobial resistance (37), leading to a broad spectrum of clinical infections, particularly in older patients who are often more susceptible due to weakened immune systems and comorbidities (such as chronic lung disorders, diabetes, and malignant tumors) (38, 39). Highly virulent *KP* (especially those that produce *ESBL* and those with multi-drug resistance) is gradually increasing in the older population. The epidemiology of *KP* infections in geriatric care settings reveals that older patients are at increased risk for *ESBL*-producing infections (40). A network-based analysis study suggested that under-monitored settings such as long-term care facilities may serve as critical nodes for *KP* transmission (41). Meanwhile, research on antibiotic resistance in older patients with UTIs showed that *KP* resistance in nursing homes was similar to hospitals, indicating a comparable resistance burden (42). Another study reported that older nursing home residents have a 40% higher risk of antibiotic-resistant Enterobacteriaceae than those in the community (43). As the global population continues to age, the number of long-term care facilities is expected to rise, which will inevitably exacerbate the burden of *KP* infections. Notably, the DALYs and death rates associated with *KP* infections in Central Europe, Eastern Europe, and Central Asia had exhibited significant upward trends in all age groups older than 15 years, especially in individuals aged over 70 years. Besides antibiotic resistance and population aging, the improper use of antibiotics and the evolution of bacterial strains (particularly the high-risk clones ST258 and ST11) also contribute significantly to the burden of diseases in these areas (44, 45). And global migration accelerates *KP* transmission, with increased cases in countries receiving immigrants. In some European nations, crowded and unhygienic conditions for refugees exacerbate *KP* spread (46). These findings emphasize the importance of improving antibiotic management and monitoring drug resistance as key strategies. And preventive measures should be taken for high-risk groups, such as identifying high-risk groups, strengthening monitor the evolution of *KP* genome, and developing vaccines prevent *KP* infection.

This study is subject to certain limitations. Firstly, the GBD data may depend heavily on comprehensive statistical models due to the inconsistent quality of data, particularly in countries with limited raw data availability. These models have inherent limitations that have been discussed in other studies (47). Secondly, the GBD framework incorporates country-level data where available, utilizing advanced modeling techniques to provide accurate global burden estimates. But validating these estimates with country-level surveillance data remains challenging. To improve future estimates, we recommend strengthening the validation process with more robust country-level surveillance data. These efforts will help address current limitations and enhance the reliability of global health assessments. Finally, the underdeveloped state of economic and medical infrastructure in less developed regions poses significant challenges in diagnosing infections caused by *KP*, leading to an underestimated disease burden. Yadav et al. (48) reported that fewer than half of the hospitals in 10 low- and middle-income countries (LMICs) possessed the capability to conduct Gram staining. The GBD 2019 Antimicrobial Resistance Collaborators speculated that even fewer hospitals in these contexts could perform cultures and susceptibility testing (13). However, many LMICs lack systematic surveillance, hindering effective monitoring of these strains. Establishing regional AMR surveillance networks and databases is essential for reducing *KP* infections. To better prevent and control outbreaks, epidemiological surveillance must become more intelligent and precise, moving from passive to proactive monitoring. This involves using artificial intelligence and machine learning to predict infection risks and understand transmission, allowing for precise resource allocation and preemptive interventions (49).

## Conclusion

This analysis found the persistent yet declining global burden of *KP* infection in the lower respiratory tract over the past three decades, evidenced by decreasing trends in ASR-DALYs and ASDRs. Nevertheless, significant disparities remain across regions, with low SDI regions, particularly sub-Saharan Africa, still experiencing the highest burden. Concurrently, the notable increase in DALYs and mortality rates among the older population in Central Europe, Eastern Europe, and Central Asia highlights emerging challenges related to inappropriate antibiotic use, widespread antimicrobial resistance, emerging virulent and multidrug-resistant strains, and an aging population. These findings emphasize the necessity for enhanced surveillance, targeted prevention strategies, and resource allocation tailored to high-risk populations and regions to mitigate the global impact of *KP* infections.

## Data Availability

The original contributions presented in the study are included in the article/Supplementary material, further inquiries can be directed to the corresponding author.

## Footnotes

^1^ http://www.healthdata.org/

## Glossary

***KP***: *Klebsiella pneumoniae*
ASR-DALYs: age-standardized disability-adjusted life years
ASDRs: age-standardized death rates
DALYs: disability-adjusted life years
YLDs: years lived with disability
YLLs: years of life lost
AAPC: average annual percentage change
GBD: Global Burden of Disease
SDI: sociodemographic index
95% UI: 95% uncertainty intervals
95% CI: 95% confidence interval
LMICs: low- and middle-income countries
HAP: hospital-acquired pneumonia
CAP: community-acquired pneumonia
UTIs: urinary tract infection
ESBL: extended-spectrum *β*-lactamase
ESBL-KP: extended-spectrum *β*-lactamase-producing *Klebsiella pneumoniae*
CRKP: carbapenem-resistant *Klebsiella pneumoniae*
CODEm: cause of death ensemble model
SDGs: sustainable development goals
IHME: Institute for Health Metrics and Evaluation
AMR: antimicrobial resistance
