# Supplementary material for: Global burden and trends of Klebsiella pneumoniae infection, 1990–2021: insights from the global burden of disease study
Source: Front Public Health. 2025 Oct 23;13:1630262. doi: 10.3389/fpubh.2025.1630262 (PMC12588909; doi:10.3389/fpubh.2025.1630262)
Supplement: Supplementary file 1 [file Supplementary_file_1.docx]

**Supplementary Table 1 The AAPCs of DALYs and death rates of *KP* infection among SDI quintiles in different age groups from 1990 to 2021.**

| **Measures** | **Locations** | **Age groups** | **AAPCs (95%CI)** |
| --- | --- | --- | --- |
| **DALYs** | High SDI | <5 years | -5.72 (-5.78 to -5.64) ^*^ |
|  |  | 5-14 years | -4.33 (-4.47 to -4.17) ^*^ |
|  |  | 15-49 years | -1.65 (-1.74 to -1.53) ^*^ |
|  |  | 50-69 years | -1.53 (-1.64 to -1.37) ^*^ |
|  |  | 70+ years | -1.81 (-1.91 to -1.68) ^*^ |
|  | High-middle SDI | <5 years | -7.93 (-8.02 to -7.85) ^*^ |
|  |  | 5-14 years | -4.86 (-5 to -4.74) ^*^ |
|  |  | 15-49 years | -1.19 (-1.33 to -1.02) ^*^ |
|  |  | 50-69 years | -0.94 (-1.07 to -0.79) ^*^ |
|  |  | 70+ years | -0.4 (-0.5 to -0.3) ^*^ |
|  | Middle SDI | <5 years | -5.91 (-5.96 to -5.84) ^*^ |
|  |  | 5-14 years | -3.85 (-3.91 to -3.77) ^*^ |
|  |  | 15-49 years | -1.7 (-1.79 to -1.63) ^*^ |
|  |  | 50-69 years | -1.25 (-1.28 to -1.22) ^*^ |
|  |  | 70+ years | -0.97 (-1 to -0.95) ^*^ |
|  | Low-middle SDI | <5 years | -4.49 (-4.55 to -4.43) ^*^ |
|  |  | 5-14 years | -3.29 (-3.37 to -3.22) ^*^ |
|  |  | 15-49 years | -1.55 (-1.59 to -1.51) ^*^ |
|  |  | 50-69 years | -1.08 (-1.13 to -1.04) ^*^ |
|  |  | 70+ years | -0.38 (-0.46 to -0.3) ^*^ |
|  | Low SDI | <5 years | -4.19 (-4.22 to -4.16) ^*^ |
|  |  | 5-14 years | -2.66 (-2.72 to -2.6) ^*^ |
|  |  | 15-49 years | -1.62 (-1.65 to -1.59) ^*^ |
|  |  | 50-69 years | -1.51 (-1.53 to -1.49) ^*^ |
|  |  | 70+ years | -1 (-1.03 to -0.97) ^*^ |
| **Death rates** | High SDI | <5 years | -5.72 (-5.79 to -5.64) ^*^ |
|  |  | 5-14 years | -4.42 (-4.54 to -4.26) ^*^ |
|  |  | 15-49 years | -1.46 (-1.55 to -1.34) ^*^ |
|  |  | 50-69 years | -1.59 (-1.74 to -1.45) ^*^ |
|  |  | 70+ years | -1.58 (-1.78 to -1.42) ^*^ |
|  | High-middle SDI | <5 years | -7.94 (-8.03 to -7.86) ^*^ |
|  |  | 5-14 years | -4.89 (-5.05 to -4.74) ^*^ |
|  |  | 15-49 years | -0.83 (-0.98 to -0.66) ^*^ |
|  |  | 50-69 years | -0.84 (-0.96 to -0.68) ^*^ |
|  |  | 70+ years | -0.08 (-0.18 to 0.02) |
|  | Middle SDI | <5 years | -5.91 (-5.97 to -5.85) ^*^ |
|  |  | 5-14 years | -3.82 (-3.89 to -3.75) ^*^ |
|  |  | 15-49 years | -1.46 (-1.54 to -1.38) ^*^ |
|  |  | 50-69 years | -1.2 (-1.23 to -1.17) ^*^ |
|  |  | 70+ years | -0.72 (-0.75 to -0.7) ^*^ |
|  | Low-middle SDI | <5 years | -4.49 (-4.55 to -4.44) ^*^ |
|  |  | 5-14 years | -3.28 (-3.36 to -3.2) ^*^ |
|  |  | 15-49 years | -1.42 (-1.46 to -1.39) ^*^ |
|  |  | 50-69 years | -1.04 (-1.11 to -0.98) ^*^ |
|  |  | 70+ years | -0.19 (-0.27 to -0.11) ^*^ |
|  | Low SDI | <5 years | -4.2 (-4.23 to -4.17) ^*^ |
|  |  | 5-14 years | -2.66 (-2.72 to -2.6) ^*^ |
|  |  | 15-49 years | -1.6 (-1.63 to -1.57) ^*^ |
|  |  | 50-69 years | -1.51 (-1.52 to -1.49) ^*^ |
|  |  | 70+ years | -0.8 (-0.83 to -0.76) ^*^ |

* Asterisk (*) indicate *P* < 0.05.

*KP*, *Klebsiella pneumoniae*; DALYs, disability-adjusted life years; AAPCs, average annual percentage changes; SDI, sociodemographic index.

**Supplementary Table 2 The AAPCs of DALYs and death rates of *KP* infection among super regions in different age groups from 1990 to 2021.**

| **Measures** | **Location** | **Age groups** | **AAPCs (95%CI)** |
| --- | --- | --- | --- |
| **DALYs** | North Africa and Middle East | <5 years | -5.96 (-6.05 to -5.89) ^*^ |
|  |  | 5-14 years | -3.86 (-3.93 to -3.8) ^*^ |
|  |  | 15-49 years | -1.78 (-1.81 to -1.75) ^*^ |
|  |  | 50-69 years | -1.46 (-1.5 to -1.43) ^*^ |
|  |  | 70+ years | -0.75 (-0.8 to -0.7) ^*^ |
|  | Sub-Saharan Africa | <5 years | -4.1 (-4.13 to -4.07) ^*^ |
|  |  | 5-14 years | -2.22 (-2.24 to -2.2) ^*^ |
|  |  | 15-49 years | -1.4 (-1.42 to -1.37) ^*^ |
|  |  | 50-69 years | -1.22 (-1.23 to -1.2) ^*^ |
|  |  | 70+ years | -0.67 (-0.69 to -0.65) ^*^ |
|  | Central Europe, Eastern Europe and Central Asia | <5 years | -3.91 (-3.98 to -3.83) ^*^ |
|  |  | 5-14 years | -2.06 (-2.12 to -1.99) ^*^ |
|  |  | 15-49 years | 0.35 (0.15 to 0.55) ^*^ |
|  |  | 50-69 years | 0.82 (0.62 to 1.04) ^*^ |
|  |  | 70+ years | 0.85 (0.64 to 1.05) ^*^ |
|  | Latin America and Caribbean | <5 years | -5.59 (-5.68 to -5.51) ^*^ |
|  |  | 5-14 years | -3.73 (-3.82 to -3.64) ^*^ |
|  |  | 15-49 years | -1.27 (-1.44 to -1.13) ^*^ |
|  |  | 50-69 years | -0.54 (-0.64 to -0.43) ^*^ |
|  |  | 70+ years | -0.55 (-0.64 to -0.47) ^*^ |
|  | Southeast Asia, East Asia and Oceania | <5 years | -6.36 (-6.4 to -6.32) ^*^ |
|  |  | 5-14 years | -4.43 (-4.51 to -4.34) ^*^ |
|  |  | 15-49 years | -2.25 (-2.29 to -2.21) ^*^ |
|  |  | 50-69 years | -2.27 (-2.31 to -2.23) ^*^ |
|  |  | 70+ years | -1.56 (-1.63 to -1.49) ^*^ |
|  | South Asia | <5 years | -4.38 (-4.48 to -4.29) ^*^ |
|  |  | 5-14 years | -4.14 (-4.39 to -3.89) ^*^ |
|  |  | 15-49 years | -2.28 (-2.38 to -2.21) ^*^ |
|  |  | 50-69 years | -1.41 (-1.48 to -1.34) ^*^ |
|  |  | 70+ years | -0.49 (-0.62 to -0.37) ^*^ |
|  | High-income | <5 years | -5 (-5.33 to -4.59) ^*^ |
|  |  | 5-14 years | -3.89 (-4.05 to -3.69) ^*^ |
|  |  | 15-49 years | -1.87 (-2.02 to -1.74) ^*^ |
|  |  | 50-69 years | -1.24 (-1.35 to -1.09) ^*^ |
|  |  | 70+ years | -1.63 (-1.76 to -1.53) ^*^ |
| **Death rates** | North Africa and Middle East | <5 years | -5.97 (-6.05 to -5.89) ^*^ |
|  |  | 5-14 years | -3.95 (-4.01 to -3.88) ^*^ |
|  |  | 15-49 years | -1.61 (-1.64 to -1.57) ^*^ |
|  |  | 50-69 years | -1.45 (-1.49 to -1.42) ^*^ |
|  |  | 70+ years | -0.57 (-0.63 to -0.53) ^*^ |
|  | Sub-Saharan Africa | <5 years | -4.1 (-4.13 to -4.07) ^*^ |
|  |  | 5-14 years | -2.22 (-2.24 to -2.19) ^*^ |
|  |  | 15-49 years | -1.36 (-1.39 to -1.34) ^*^ |
|  |  | 50-69 years | -1.24 (-1.26 to -1.23) ^*^ |
|  |  | 70+ years | -0.48 (-0.5 to -0.46) ^*^ |
|  | Central Europe, Eastern Europe and Central Asia | <5 years | -3.91 (-3.99 to -3.83) ^*^ |
|  |  | 5-14 years | -2.03 (-2.09 to -1.96) ^*^ |
|  |  | 15-49 years | 0.63 (0.41 to 0.87) ^*^ |
|  |  | 50-69 years | 0.92 (0.7 to 1.18) ^*^ |
|  |  | 70+ years | 1.00 (0.85 to 1.17) ^*^ |
|  | Latin America and Caribbean | <5 years | -5.6 (-5.68 to -5.52) ^*^ |
|  |  | 5-14 years | -3.73 (-3.82 to -3.64) ^*^ |
|  |  | 15-49 years | -1.06 (-1.24 to -0.91) ^*^ |
|  |  | 50-69 years | -0.52 (-0.6 to -0.41) ^*^ |
|  |  | 70+ years | -0.42 (-0.51 to -0.32) ^*^ |
|  | Southeast Asia, East Asia and Oceania | <5 years | -6.36 (-6.41 to -6.32) ^*^ |
|  |  | 5-14 years | -4.41 (-4.5 to -4.33) ^*^ |
|  |  | 15-49 years | -1.97 (-2.01 to -1.93) ^*^ |
|  |  | 50-69 years | -2.18 (-2.22 to -2.15) ^*^ |
|  |  | 70+ years | -1.21 (-1.28 to -1.14) ^*^ |
|  | South Asia | <5 years | -4.39 (-4.48 to -4.3) ^*^ |
|  |  | 5-14 years | -4.13 (-4.39 to -3.88) ^*^ |
|  |  | 15-49 years | -2.17 (-2.29 to -2.09) ^*^ |
|  |  | 50-69 years | -1.31 (-1.41 to -1.23) ^*^ |
|  |  | 70+ years | -0.33 (-0.45 to -0.21) ^*^ |
|  | High-income | <5 years | -5.01 (-5.34 to -4.59) ^*^ |
|  |  | 5-14 years | -3.87 (-4.03 to -3.67) ^*^ |
|  |  | 15-49 years | -1.7 (-1.84 to -1.57) ^*^ |
|  |  | 50-69 years | -1.4 (-1.52 to -1.28) ^*^ |
|  |  | 70+ years | -1.3 (-1.43 to -1.19) ^*^ |

* Asterisk (*) indicate *P* < 0.05.

*KP*, *Klebsiella pneumoniae*; DALYs, disability-adjusted life years; AAPCs, average annual percentage changes.

**
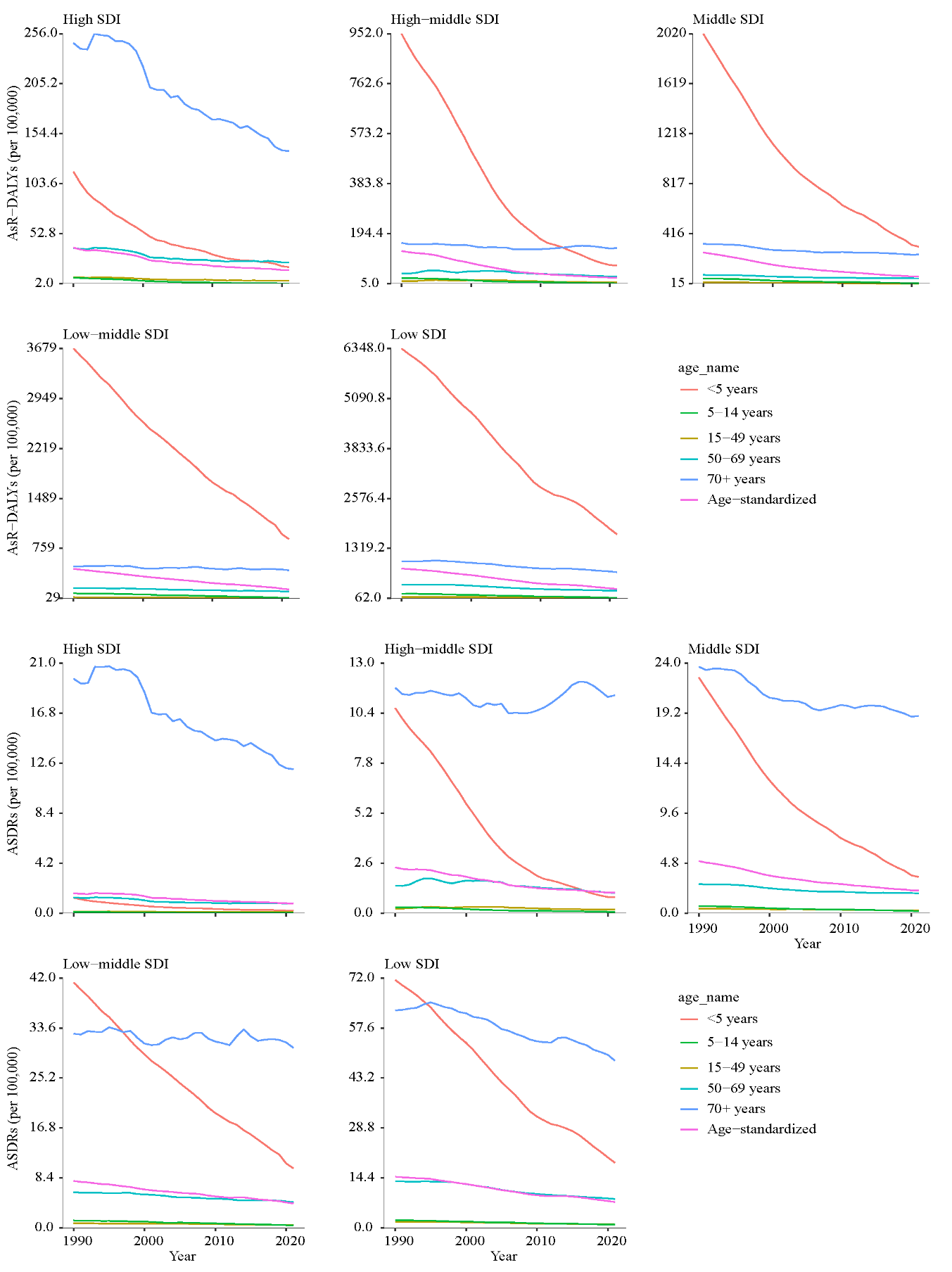
**

**Supplementary Figure 1 The AAPCs of ASR-DALYs and ASDRs of *KP* infection among SDI quintiles in different age groups from 1990 to 2021.**

*KP*, *Klebsiella pneumoniae*; ASR-DALYs, age-standardized disability-adjusted life years; ASDR, age-standardized death rate; AAPCs, average annual percentage changes; SDI, sociodemographic index.

**
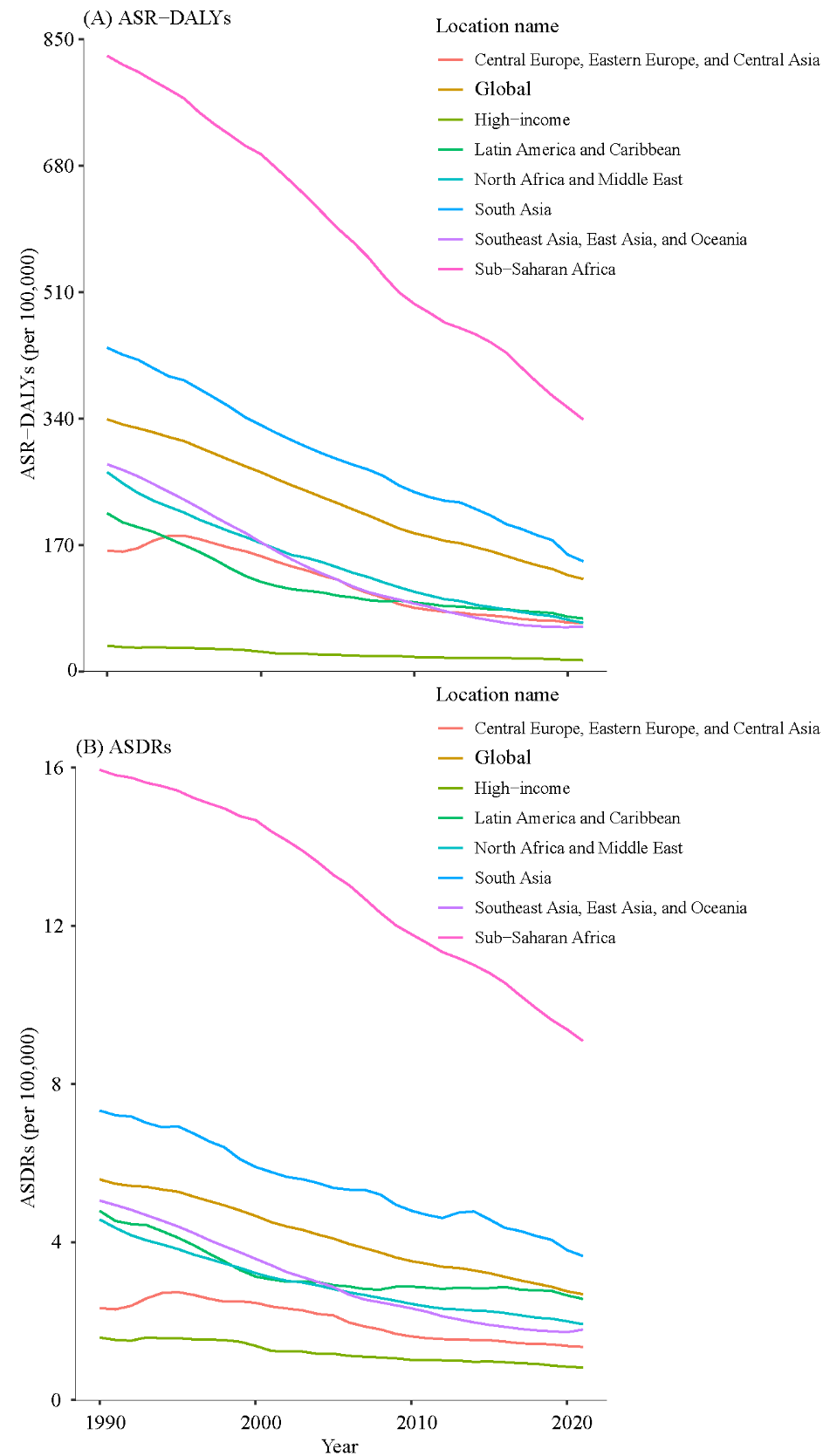
**

**Supplementary Figure 2 The AAPCs of ASR-DALYs and ASDRs of *KP* infection among super regions from 1990 to 2021.** (A) ASR-DALYs (B) ASDRs.

*KP*, *Klebsiella pneumoniae*; ASR-DALYs, age-standardized disability-adjusted life years; ASDR, age-standardized death rate; AAPCs, average annual percentage changes.
